# Mild COVID-19 Illness as a Risk Factor for Venous Thromboembolism

**DOI:** 10.7759/cureus.18236

**Published:** 2021-09-24

**Authors:** María Manuela Clavijo, María de los Angeles Vicente Reparaz, Juan I Ruiz, María Angeles Acuña, Claudia E Casali, María Florencia Aizpurua, Carolina V Mahuad, Sebastian Eciolaza, Adriana Ventura, Gonzalo M Garate

**Affiliations:** 1 Hematology and Oncology, Hospital Aleman, Buenos Aires, ARG; 2 Internal Medicine, Hospital Aleman, Buenos Aires, ARG; 3 Hematology, Hospital Aleman, Buenos Aires, ARG

**Keywords:** coronavirus disease 2019 (covid-19), venous thromboembolism (vte), pulmonary embolism, deep vein thrombosis (dvt), risk factor for vte, mild covid-19 infection

## Abstract

Introduction

From the beginning of the current coronavirus disease 2019 (COVID-19) pandemic, there is cumulative evidence suggesting that patients hospitalized due to this disease are at a high risk for venous thromboembolism (VTE). The association between mild non-hospitalized illness and VTE is unclear. The purpose of this research is to assess the association between VTE and mild COVID-19 infection.

Methods

A case-control study was conducted. The cases were adult patients diagnosed with VTE from March 1, 2020 to March 31, 2021. The controls were randomly chosen adult patients who required healthcare services that were equivalent to those of the cases, for any cause, during the same time period, without a VTE diagnosis. To assess the association between mild COVID and VTE, a multivariate logistic regression analysis was conducted, considering other thromboembolic risk variables, such as age, gender and active cancer, among others. A p-value <0.05 was considered statistically significant.

Results

A total of 186 cases and 475 controls were analyzed. There were 21 (11.3%) and 31 (6.5%) patients infected with mild COVID-19 in the previous three months in the groups of cases and controls, respectively. Mild COVID-19 infection was statistically significant as a risk factor for VTE both in the univariate analysis and in the multivariate analysis, OR=1.82 (95% CI 1.02-3.26) and OR=2.62 (95% CI 1.34-5.13), respectively.

Conclusion

Mild COVID-19 infection might be an independent risk factor for VTE. We conclude that the results suggest some thromboprophylaxis strategy should be considered in certain patients with COVID-19 infection in an outpatient fashion.

## Introduction

Coronavirus disease 2019 (COVID-19), a viral illness caused by the severe acute respiratory syndrome coronavirus-2 (SARS-CoV-2), results in respiratory pathology and extrapulmonary manifestations [1]. VTE is a known cause of morbidity and mortality both in outpatient and inpatient settings, among patients hospitalized for medical or surgical reasons. From the beginning of the current COVID-19 pandemic, there is cumulative evidence suggesting that patients hospitalized due to this disease are at a high risk for VTE, including those who have been prescribed pharmacological thromboprophylaxis according to the international guidelines on patients hospitalized for other non-surgical causes [1]. Furthermore, there is a high variability in reports regarding VTE in patients hospitalized for COVID-19 and no consensus has been reached among different scientific societies as to the dosing and duration of anticoagulant therapy in these patients.

A meta-analysis published in 2020 reported a global incidence of VTE in hospitalized patients of 14.1% (95% CI 11.6-16.9) [2]. These results were higher than those reported in trials conducted prior to the current pandemic, in which a 2.8%-5.6% incidence of VTE was found in patients hospitalized for non-surgical causes [3]. The American Society of Hematology (ASH) recommends the use of prophylactic-intensity anticoagulation over intermediate-intensity or therapeutic-intensity anticoagulation in patients hospitalized for COVID-19, based on very low certainty in the evidence [4]. Reports on VTE and COVID-19 are centered upon hospitalized patients, especially those with a moderate to severe illness. However, evidence regarding the association between VTE and mild COVID-19 infection is unclear. The National Institutes of Health (NIH) recommend against the use of thromboprophylaxis in non-hospitalized patients with COVID-19 infection, based on expert opinion [3].

In the literature, we have found various case reports that refer to the association between VTE and patients with mild COVID-19 illness, most of whom were treated on an outpatient basis [5-13]. In the daily practice, pharmacological thromboprophylaxis is frequently used in patients with mild disease who have another risk factor for increased blood clotting. Nevertheless, this practice lacks strong scientific grounding and is not recommended in the clinical practice guidelines. The primary objective of this research is to assess whether mild COVID-19 illness is an independent risk factor for VTE during the three months following infection and to estimate the magnitude of such risk.

## Materials and methods

A case-control study was conducted. The cases were adult patients with any of the following problems in their medical records: venous thrombosis, deep vein thrombosis, thromboembolic disease, venous thromboembolism or pulmonary thromboembolism during the period between March 1, 2020 and March 31, 2021. The diagnosis of VTE was confirmed by Doppler ultrasound or CT scan after suspicion according to each physicians’ criteria or incidentally during examination for other disorders. 

The controls were defined as adult patients who required healthcare services that were equivalent to those of the cases, for any cause, during the same time period, and who were not diagnosed with VTE. A randomized sample of two to three times the number of cases was requested.

Patients were identified retrospectively through the database of a community hospital. Recruitment was from patients who were attended by physicians as outpatient at the emergency department, hematology, oncology, traumatology, pulmonology, cardiology or phlebology. Those patients with catheter-associated thrombosis or who were receiving anticoagulation for any cause were excluded.

Mild COVID-19 illness was defined as a diagnosis of COVID-19 infection through a polymerase chain reaction test via nasopharyngeal swab in patients who came to the healthcare facility for symptoms compatible with the disease (and met the definition of suspicious COVID-19 case according to Argentinean regulations) [14] and who did not have pneumonia or had mild pneumonia without need for oxygen therapy.

For the association analysis, VTE was established as the dependent variable, whereas the independent variables were mild COVID-19 disease during the three months prior to the VTE diagnosis or to the consultation for any cause and the variables considered for the multivariate analysis. The latter are as follows: male gender, obesity, smoking status, pregnancy, all-cause hospitalization in the previous three months, major surgery in the previous three months, history of VTE, known acquired or inherited thrombophilia, active cancer, polytrauma in the previous three months and hormone therapy.

The protocol was approved by the Ethics and Research Committee of Hospital Aleman (CEIHA).

The logistic regression model was used. For the multivariate analysis, variables with a p-value <0.1 were contemplated. A p-value <0.05 was considered statistically significant.

For the statistical analysis, STATA version 13 was used [15].

## Results

The study analyzed 661 patients, 186 cases, and 475 controls. The average age was 53.99 years (SD 19.62). The average age of the cases and controls was 64.80 (SD 16.21) and 49.75 years (SD 19.22), respectively (p<0.01). Male patients accounted for 41.9% of the population, 49% of the cases and 39% of the controls (p=0.02).

Out of the 661 patients, 52 had mild COVID-19 infection, and 4 (7.6%) of those were hospitalized. All these hospitalizations were related to the COVID-19 infection, 1 because of personal difficulty of home isolation, 2 because of advanced age and comorbidities and 1 for cardiology monitoring because of transitory sinus bradycardia.

Within the group of cases, there were 21 (11.3%) patients with mild COVID-19 disease in the previous three months, 7 (3.7%) patients with obesity, 38 (20.4%) patients with smoking status, 0 pregnant women, 72 (38.7%) patients with all-cause hospitalization in the previous three months, 29 (15%) patients with major surgery in the previous three months, 20 (10.7%) patients with history of VTE, 3 (1.9%) patients with known thrombophilia, 57 (30.6%) patients with active cancer, 5 (2.7%) patients with polytrauma in the previous three months and 5 (2.7%) patients under hormone therapy. (TABLE 1)

Regarding risk factors for VTE in the case group, out of 21 patients with mild COVID-19 disease and VTE, 10 (48%) had at least one other major risk factor for VTE (3 history of VTE, 3 active cancer, 1 obesity, 3 smokers, 2 major surgery in the previous three months, 5 all-cause hospitalization in the previous three months), 5 out of 10 had at least two of these factors. The rest of the patients with mild COVID-19 disease and VTE (11 patients), 9 (82%) were male, two of them had overweight (body mass index between 26 and 29).

The following VTE events were detected: pulmonary thromboembolism (PTE) in 65 patients (35%), below-knee deep venous thrombosis (DVT) in 59 (31.7%), DVT in an unusual site in 9 (4.8%), above-knee DVT in 29 (15%), DVT/PTE in 24 (12.9%). Within the group of patients who have had a mild COVID-19 disease (21 patients), the events were as follows: 4 PTEs, 8 below-knee DVTs, 5 above-knee DVTs, 3 DVTs/PTEs, 1 DVT in an unusual site (mesenteric thrombosis). A total of 6 out of 12 patients with more severe VTE events (PTE and/or above-knee DVT) had at least one other major risk factor for VTE.

Within the group of controls, there were 31 (6.5%) patients with mild COVID-19 disease in the previous three months, 11 (2.3%) patients with obesity, 55 (11.6%) patients with smoking status, 3 (0.6%) pregnant women, 62 (13%) patients with all-cause hospitalization in the previous three months, 14 (2.9%) patients with major surgery in the previous three months, 20 (4.2%) patients with history of VTE, 2 (0.4%) patients with known thrombophilia, 53 (11.1%) patients with active cancer, 5 (1%) patients with polytrauma in the previous three months and 6 (1.3%) patients under hormone therapy (Table 1).

**Table 1:** Patient features.

N=661	Cases (n=186)	Controls (n=475)	p
Age	64.80 (SD 16.21)	49.75 (SD 19.22)	<0.01
Males	91 (49%)	186 (39%)	0.02
Mild COVID-19	21 (11.3%)	31 (6.5%)	0.04
Obesity	7 (3.7%)	11 (2.3%)	0.6
Smoking status	38 (20.4%)	55 (11.6%)	<0.01
Pregnancy	0	3 (0.6%)	0.3
All-cause hospitalization in the previous three months	72 (38.7%)	62 (13%)	<0.01
Major surgery in the previous three months	29 (15%)	14 (2.9%)	<0.01
History of VTE	20 (10.7%)	20 (4.2%)	<0.01
Known thrombophilia	3 (1.9%)	2 (0.4%)	0.1
Active cancer	57 (30.6%)	53 (11.1%)	<0.01
Polytrauma in the previous three months	5 (2.7%)	5 (1%)	0.1
Hormone therapy	5 (2.7%)	6 (1.3%)	0.2

The variables that were considered significant for inclusion in the multivariate analysis were: age, male gender, history of VTE, smoking status, major surgery in the previous three months, active cancer and all-cause hospitalization in the previous three months.

Mild COVID-19 disease was statistically significant as a risk factor for VTE both in the univariate analysis and in the multivariate analysis, OR=1.82 (95% CI 1.02-3.26) and OR=2.62 (95% CI 1.34-5.13), respectively (Table 2).

**Table 2:** Multivariate analysis.

Risk factor	OR	95% CI	p
Mild COVID-19 in the previous three months	2.63	1.34-5.13	<0.01
Age	1.03	1.02-1.04	<0.01
Males	1.45	0.98-2.14	0.062
History of VTE	1.95	0.95-3.98	0.066
Smoking status	1.15	0.68-1.96	0.584
Major surgery in the previous three months	1.86	0.84-4.13	0.125
Active cancer	2.11	1.31-3.39	<0.01
All-cause hospitalization within the previous three months	2.29	1.40-3.74	<0.01

## Discussion

Mild COVID-19 disease might be an independent risk factor for VTE in the three months following the infection.

The magnitude of the association in this article was comparable to and even higher than the magnitude for known risk factors, such as active cancer or recent hospitalization. In this study, 48% of the patients with COVID-19 disease and VTE had also at least one other major risk factor for VTE.

The mechanism through which the COVID-19 infection generates thromboembolic events has been described in multiple papers. Among the processes described, we may find an imbalance in the renin-angiotensin-aldosterone system homeostasis caused by the downregulation of aminopeptidase ACE2, signaling mechanisms subsequent to cell apoptosis, development of endotheliitis, hyperinflammatory state, formation of neutrophil extracellular traps, etc. [16].

Several studies have assessed inflammatory parameters in patients diagnosed with COVID-19 infection [16-20]. Fu et al. [17] assessed differences in parameters in patients with mild or moderate illness as compared to patients with severe illness. As it might be expected, all parameters under assessment were significantly higher in patients with severe illness than in those with mild or moderate infection, although the patients in the latter group had some increased parameters as well, such as C-reactive protein [17].

Our paper shows an association between mild COVID-19 illness and VTE. As this is a case-control study, the incidence of VTE in the population with mild COVID-19 may not be established. Considering the mechanisms described, we assume that this incidence is far lower than the one reported in hospitalized patients with moderate to severe inflammatory manifestations.

In the literature, there are various papers on VTE in patients with mild COVID-19 illness: a report on four cases, including three PTEs and one lower-limb DVT; three reports of thrombosis in an unusual site (two cases of cerebral thrombosis, one case of mesenteric thrombosis); one case of DVT/PTE; one case of DVT; one case of massive non-fatal PTE; one case of fatal PTE following asymptomatic COVID‑19 infection; and one case of a patient with DVT and APS [5-13]. Our research found lower‑limb DVT most frequently, followed by PTE, PTE/DVT, and there was one case of mesenteric thrombosis in a young patient diagnosed with mild COVID-19 infection 38 days before the VTE diagnosis.

In this study, all patients with VTE who have had mild COVID-19 disease experienced VTE symptoms that made them go to the healthcare facility. Chen et al. [21] published a high incidence of incidental VTE in patients diagnosed with mild or moderate COVID-19 disease who had screening tests performed. They found a VTE prevalence of 82.6% [21].

Moreover, arterial events have also been reported in patients with mild COVID-19 disease [22-26], as well as apparently idiopathic VTE in patients that were diagnosed with COVID-19 through screening tests [27].

These findings support the assumption that mild COVID-19 disease might be a predisposing factor for VTE.

Nevertheless, the weaknesses of this study are the fact that it is confined to a single site with a small sample of COVID-19 patients and that it is retrospective, which involves a potential reporting bias. Additionally, some cases of COVID-19 infection may have been missed considering the information bias of patients who did not complain of relevant symptoms, and did not undergo testing for COVID-19 infection.

## Conclusions

Our study suggests that mild COVID-19 disease might be an independent risk factor for VTE in the three months following the infection. The results found in this study are consistent with other papers cited and support the need to consider some thromboprophylaxis strategy in certain patients with mild COVID-19 disease in outpatient settings.
